# Drama-based therapy program in the recovery of adults with addictive disorders

**DOI:** 10.1192/j.eurpsy.2024.859

**Published:** 2024-08-27

**Authors:** M. Krupa, A. Balogh-Pécsi

**Affiliations:** ^1^Department of Hungarian and Applied Linguistics, University of Szeged, Szeged; ^2^University of István Széchenyi, Győr, Hungary

## Abstract

**Introduction:**

Following the pandemic, we can find many new communication situations. Social relationships have changed a lot and are developing differently due to digital development, new lifestyles, and the effects of COVID-19. These components: social media, the transformation of interpersonal relationships, and the use of the platforms provided by the internet can lead to addictive disorders as risk factors.

**Objectives:**

In this presentation, we review studies investigating the relationship between the new digital techniques, social connection, and communication development of adults with addictive disorders. We attempt to provide a summary of new theories and the areas currently being researched around the topic. Another aim of our research is to present the new drama-based therapy theories and methods in adults with addictive disorders.

**Methods:**

To learn about recent international results, we conducted a literature search in 3 databases (PubMed, Medline, Web of Science) using the following keywords: drama therapy, addiction, emotion regulation, and adults, over the past 5 years. Empirical journal articles in English were used to prepare the literature review. Exclusion criteria were: the appearance publication before the year 2017 and the adolescent population.

**Results:**

Changes in social behavior, emotion regulation, and addictive disorder were correlated. The studies examined social communications and loneliness in primarily cross-sectional studies design. The escapism from interpersonal relations and low self-esteem is the highest motivation to start regular videogame playing or using social media without control which becomes an addictive disorder.

**Conclusions:**

Problematic social media use and changes in social connection threaten adults’ mental health. The diagnosis of emotion dysregulation, low self-esteem, and social disconnection is the detection of risk factors for addictive disorders. The new methods and tools of drama-based therapy are new prevention possibilities for these risk factors. In this way, it is a relevant issue in the field of education science.

**Disclosure of Interest:**

None Declared

